# Multi-scale fusion for RGB-D indoor semantic segmentation

**DOI:** 10.1038/s41598-022-24836-9

**Published:** 2022-11-24

**Authors:** Shiyi Jiang, Yang Xu, Danyang Li, Runze Fan

**Affiliations:** 1 College of Big Data and Information Engineering, Guizhou University, Guiyang, 550025 China; 2 Guiyang Aluminum-magnesium Design and Research Institute Co., Ltd., Guiyang, 550009 China

**Keywords:** Computational science, Information technology, Software

## Abstract

In computer vision, convolution and pooling operations tend to lose high-frequency information, and the contour details will also disappear with the deepening of the network, especially in image semantic segmentation. For RGB-D image semantic segmentation, all the effective information of RGB and depth image can not be used effectively, while the form of wavelet transform can retain the low and high frequency information of the original image perfectly. In order to solve the information losing problems, we proposed an RGB-D indoor semantic segmentation network based on multi-scale fusion: designed a wavelet transform fusion module to retain contour details, a nonsubsampled contourlet transform to replace the pooling operation, and a multiple pyramid module to aggregate multi-scale information and context global information. The proposed method can retain the characteristics of multi-scale information with the help of wavelet transform, and make full use of the complementarity of high and low frequency information. As the depth of the convolutional neural network increases without losing the multi-frequency characteristics, the segmentation accuracy of image edge contour details is also improved. We evaluated our proposed efficient method on commonly used indoor datasets NYUv2 and SUNRGB-D, and the results showed that we achieved state-of-the-art performance and real-time inference.

## Introduction

Along with the development of the computer vision field, in view of the image semantic segmentation has become an important subject in the field of image segmentation is to classify each pixel in the image and predict belongs to tags, location, through the image is divided into several of the same areas of the nature of the process to provide a complete understanding of the scenario^1^. At present, semantic segmentation has been widely applied in automatic driving^2^, remote sensing analysis^3^, medical image processing^4^, etc. As for the semantic segmentation of indoor scenes, the factors affecting the semantic segmentation of indoor scenes are quite complex (such as illumination, occlusion, etc.)^5^. Since common downsampling operations of neural networks such as Max pooling, average pooling, and convolution operations do not separate noise. In the study of indoor scenes, the main purpose is to reduce the influence of these factors and improve the accuracy of semantic segmentation. Previous deep learning approach by dealing with the indoor scene RGB image to achieve end-to-end image semantic segmentation, due to the complex indoor scene, uneven illumination, color texture repeat degrees higher, the indoor scene semantic based on RGB image segmentation is category error classification, edge false segmentation, robustness and accuracy is not high. In recent years, research has found that semantic segmentation based on RGB-D images can improve the segmentation effect through the depth information in the scene which is less affected by lighting and other conditions. At the same time, the depth information can also reflect the position and other relationships between objects, which is complementary to RGB color images and also has a certain auxiliary role in semantic segmentation. Couprie et al.^6^ found that adding depth information could improve the segmentation accuracy of similar objects.

With the emergence of depth cameras such as TOF (Time-of-flight) and Kinect, it becomes easier to obtain the depth information of scenes. However, it is always a challenging problem to find an effective and high-quality fusion method between RGB color images and depth information. Jiang et al.^7^ added and fused the RGB and depth information of the middle layer of the encoder through parallel processing. Li et al.^8^ believed that single-layer fusion could not complement the color image and depth map well, and chose to fuse features before final prediction. Chang et al.^9^ added depth information to the loss function and used the change of depth value on the edge of the classified object to constrain the network training. However, there are still many problems with the above approach: Factors effecting on the one hand, the indoor noise exists in different frequency part of image more^10^, while traditional convolution neural network down sampling operations such as average pooling, maximum pooling will not separate different frequency information, which can lead to high frequency noise increased with the increase of the depth of the network are preserved, the sampling data of aliasing in the basic structure of the residual noise destroys the image features, Thus, it brings difficulties to the image segmentation task. On the other hand, the depth image is used as the fourth channel to fuse with the color image, which does not make full use of the complementarity of RGB color information and depth information. With the increase of depth of neural network, there are problems such as multi-scale information feature loss in different downsampling methods. However, for the problem of indoor scene semantic segmentation, the efficiency of multi-scale feature extraction affects the segmentation accuracy of small target objects. Compared with the RGB image, the depth image is more sparse, which represents the depth value of each pixel in the RGB image. The edge contour information, that is, high-frequency information, can be better obtained from the depth image, but traditional convolution, pooling and other operations often lose high-frequency features. The direct splicing fusion method requires convolution operation to add a large number of calculation parameters, and the network model is too large for the neural network to become an urgent problem.

In the process of signal processing, in order to avoid aliasing features and separate information of different frequencies, time-frequency analysis tools are usually used to decompose data into different frequency intervals, such as wavelet transform^11^. In recent years, combining wavelet transformation with deep learning approach has developed rapidly, such as whether Liu et al.^12^ wavelet transform and inverse transformation to replace the network of sampling for super-resolution images, or Ramamonjisoa et al.^13^, such as using wavelet transform fusion into the network in the middle tier RGB color image processing, good results have been obtained. But simply replace wavelet transform in the network under the sampling operation did not make full use of it in the face of non-stationary information can be effectively extracted features characteristic of different scales, different frequency domain information is also different, for visual reasoning tasks, different from the traditional sampling retention under the low frequency information, high frequency information is often contains the detail^14^ the outline of the object, especially for indoor RGB-D image segmentation, noise, depth and other effects exist in different scale and frequency information. This is a big problem that cannot be solved by the above methods. Aiming at the problems existing in the RGB-D semantic segmentation method of indoor scenes. Inspired by the above methods, this paper proposes a semantic segmentation network for indoor scene RGB-D images based on multi-scale fusion (MSFNet), aiming at the existing problems in indoor scene RGB-D semantic segmentation methods, The network encoder uses Resnet-50 as the baseline, and designs a fusion Module based on wavelet transform. The original image is divided into four frequency children by wavelet transform, and image fusion is realized by inverse wavelet transform, which is respectively used for RGB color image and depth image fusion. In the deep layer of the network, the feature information of different scales is fused, and the information of different scales of the image is more refined. And different from ASPP and other related fusion modules, the wavelet transform fusion module does not add additional calculation, which is more lightweight. And the refinement on the feature resolution is more intensive and accurate. We evaluated our network on commonly used indoor datasets NYUv2^15^ and SUNRGB-D^16^ and obtained high-quality results. The main contributions of this paper are as follows:

- We propose a semantic segmentation network for indoor scene RGB-D images based on multi-scale fusion (MSFNet), aiming at the existing problems in indoor scene RGB-D semantic segmentation methods.
- A new multi-scale fusion method is proposed. Through wavelet transform and inverse wavelet transform, images of subbands with different frequencies are fused, Nonsubsampled contour wave transform replaces the pooling operation of the encoder in baseline, preserving the multi-scale properties and directionality of the original image, and designed a multiple pyramid module (MPM) to aggregate multi-scale information and context global information, retained the edge contour information of images, the number of parameters is reduced, and the network operation efficiency is accelerated.
- Extensive experiments using NYUv2 and SUN-RGBD public datasets demonstrate that our proposed method has better performance and robustness than the current mainstream methods in different data domains.

## Related work

### Semantic segmentation

In the field of computer vision, the emergence of deep learning has made up for many deficiencies in traditional methods. In 2015, Long et al.^17^ proposed Fully Convolutional Networks (FCN), which replaced the last Fully connected layer of the traditional convolutional neural network with a deconvolution layer, realized the “end-to-end” RGB color image semantic segmentation, and added the pooling layer and jump connection. Address feature loss issues. Ronneberger et al.^18^ proposed U-NET, which adopted an Encoder-decoder structure for small targets and added richer feature information fusion. Badrinarayanan et al.^19^ proposed Segmentation Network (SegNet), which adopted the same structure as U-NET. In order to prevent information loss, SegNet adopted pooling with index to solve the problem of location information loss caused by multiple pooling. Based on ResNet^20^, Zhao et al.^21^ proposed the Pyramid Scene Parsing Network (PSPNet), which introduces the Pyramid fusion module to integrate the feature information of different scales according to the prior knowledge of the context in the Scene, and solves the problem of spatial information loss in FCN. There are also recent approaches to improve segmentation accuracy by adding supervision during training, Borse et al.^22^ propose Hierarchically Supervised Semantic Segmentation (HS3), a training scheme that supervises intermediate layers in a segmentation network to learn meaningful representations by varying task complexity. Common network architectures for semantic segmentation follow an encoder-decoder design: the encoder extracts features from the input and downsamples them to reduce computational effort, the decoder upsamples, deconvolves them to recover the input resolution, and finally assigns a semantic class to each input pixel. However, there are also recent studies that combine CNN with Transformer. Li et al.^23^ proposed a dual encoding-decoding structure of the X-shaped network, integrated both characteristics of CNN and Transformer, achieves good segmentation results

### RGB-D semantic segmentation

The depth image can be used as the complement of RGB color image to provide the geometric information of the scene, so as to improve the accuracy of segmentation. However, how effectively integrate deep information into network training is still a challenge. The fusion method can be classified into three types: early fusion, middle fusion and late fusion. Some of the early methods directly the depth image to the RGB color channels^7,24,25^, and under the assumption that there are four channel input RGB-D data in the training, but the depth of information as a color image directly between the fourth channel is not very good complementarity, training and the use of two branches of the network, one for RGB color images, One is used for depth information image, and the two are fused in the middle layer to get good results. In this way, each branch can extract its own features and use them for fusion, such as color and texture from RGB images, geometry, and location information independent of lighting from depth images. Hazirbas et al.^26^ proposed FuseNet, which used two branches to extract features from RGB and depth images at the same time, and fused the depth features into RGB feature maps with the deepening of the network. Hu et al.^27^ proposed ACNet to fuse the features extracted from RGB and depth images on the third encoder branch and added the attention module. In the middle of fusion, people begin to pay attention to the fusion in different stages, and explore different fusion methods. Gupta et al.^28^ proposed the Horizontal dimensions, Height above ground, Angle of the surface normal (HHA) depth information representation. The depth image is converted into different channels (horizontal difference, ground height and surface normal vector Angle), but HHA only emphasizes the complementary information between the data of each channel but ignores its independence, and it is computatively expensive. At present, more and more research focuses on changing the fusion efficiency and using different levels of information. Xing et al.^29^ propose a novel method to effectively integrate RGB and HHA features By replacing identity mappings in Resnet-based two-stream network with idempotent Mappings. Chen et al.^30^ proposed Spatial Information Guided Convolution (S-CONV), which effectively integrates RGB features with 3D Spatial Information. However, in the process of feature fusion, both convolution operation and pooling operation are accompanied by the loss of feature information, and the complementarity of RGB color image and depth image cannot be fully utilized. Most existing methods exploit a multi-stage fusion strategy to propagate depth feature to the RGB branch. However, at the very deep stage, the propagation in a simple element-wise addition manner can not fully utilize the depth information. Chen et al.^31^ proposed Global-Local propagation network (GLPNet) to solve this problem. they used a local context fusion module (L-CFM) to dynamically align both modalities before element-wise fusion, and designed a global context fusion module (G-CFM) to propagate the depth information to the RGB branch by jointly modeling the multi-modal global context features. Cao et al.^32^ introduced a Shape-aware Convolutional layer (ShapeConv) for processing the depth feature, where the depth feature is firstly decomposed into a shape-component and a base-component, next two learnable weights are introduced to cooperate with them independently, and finally a convolution is applied on the re-weighted combination of these two components.. This operation need to consume large amounts of computing resources, however, is not conducive to deploy on mobile equipment, combined with the RGB-D image fusion problem, according to the characteristics of the wavelet transform, we will be combined with neural network, wavelet transform for image processing and fusion of the different frequencies by multi-scale method to improve the effect of color image and the depth of the image fusion, And because of the characteristics of wavelet transform, it does not consume additional computing resources.

### Wavelet transform in computer vision

Wavelet transform is often used in signal processing and image analysis because of its multi-resolution analysis and stepwise decomposition. At the same time, the multi-scale decomposition of wavelet transform is more consistent with human vision mechanism. In neural networks, both convolution operation and pooling operation (maximum pooling, average pooling) are lost in different frequency information to a certain extent, and the information features of different frequencies can be retained by combining with wavelet transform. Bae et al.^33^ combined wavelet transform with residual network and found that more subbands of wavelet transform could improve the learning effect of network. Guo et al.^34^ proposed deep Wavelet Super Resolution to improve network performance by processing the missing details in the process of subband restoration. Li et al.^35^ proposed to replace the pooling operation in neural network with wavelet transform, which could retain the high-frequency information and edge details of the original image. Li et al.^36^ used wavelet transform and inverse wavelet transform instead of downsampling and upsampling operations in U-Net. Ramamonjisoa et al.^37^ integrate wavelet transforms into the encoder-decoder process, requiring less than half the multiply adds in the decoder network. Wavelet transform instead of down sampling operation, however, does not take full advantage of image multi-scale, frequency characteristics, more is not necessary to use wavelet transform to simply reduce the resolution of the image, using the wavelet transform can keep the different scales of information fusion, especially in the depth of the image and high frequency and low frequency characteristics of the color image is not the same, Based on the characteristics of the above wavelet transform, we propose a multi-scale fusion RGB-D indoor image segmentation network, which integrates the low-frequency and high-frequency features of the original image without adding extra computation.

## Proposed method

**Figure 1:**
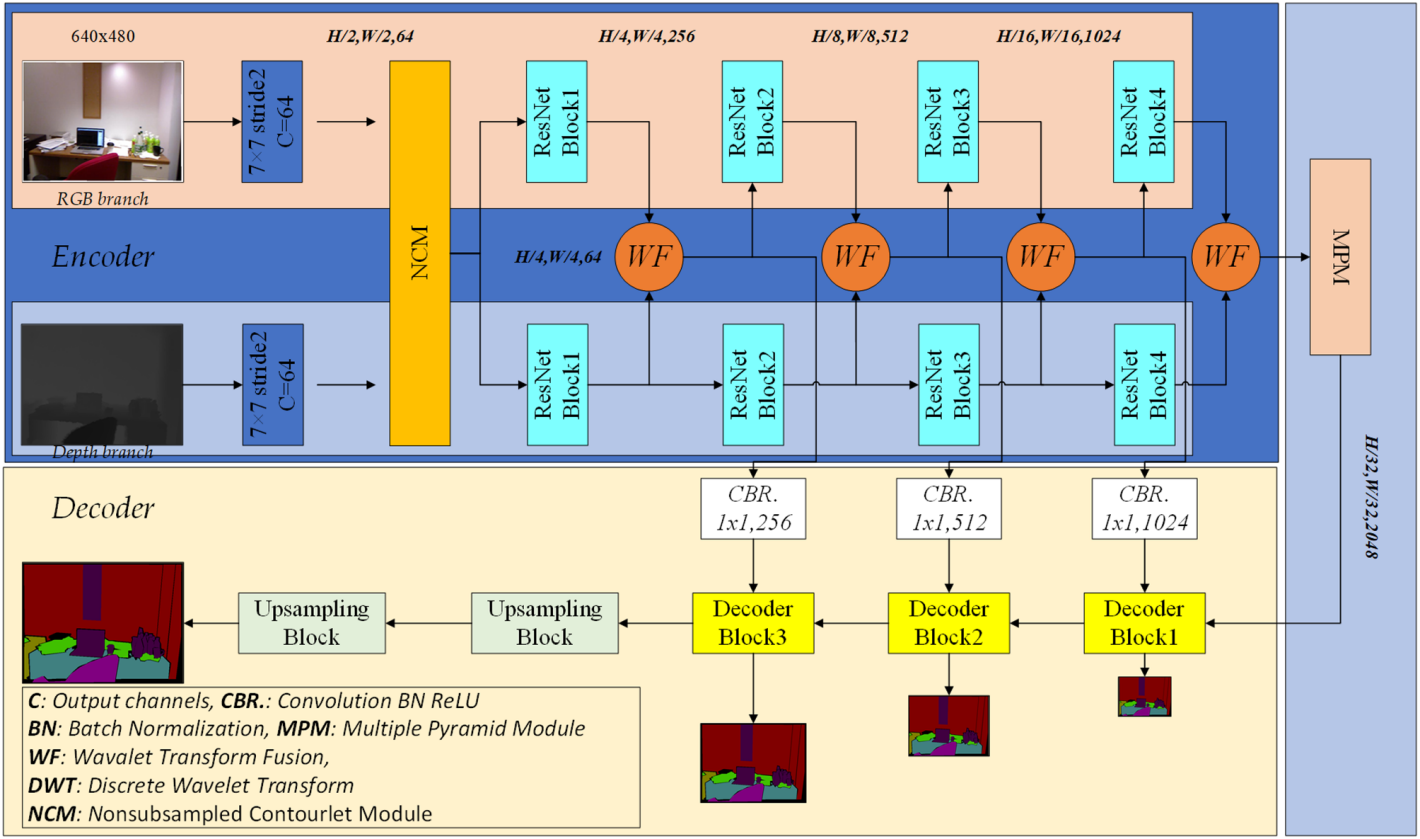
Network structure of model.

In this paper, we adopted the “encoder-decoder” structure, and designed the wavelet transform fusion module. The nonsubsampled contourlet wave transform is used to replace the pooling operation and the inverse wavelet transform to improve the semantic segmentation accuracy. The encoder network adopts Resnet-50 as the backbone network, and removes the fully connected layer. There are two branches in the network to extract the RGB and depth feature information in the original image respectively. At the same time, the feature fusion of RGB-D is carried out through the wavelet transform fusion module. At the connection between the encoder and the decoder, we designed a multiple pyramid module to aggregate feature information and global context information at different scales. Finally, the above features are up-sampled several times through the decoder network. Each module of the decoder upsamples the features by a factor of two, and performs better feature mapping by convolution and connection of the encoder. Then, the high-resolution images are gradually recovered and the semantic segmentation results are output. The network model structure is shown in Fig. 1.

### Wavelet transform

In this part, we first introduce 2D Wavelet Transform. We select the efficient and fast Haar Wavelet, then introduce the designed Wavelet Transform fusion module.

The original image X can be decomposed into four subband images by using the discrete wavelet transform (DWT)^38^ of 2D Haar wavelet transform, it can decompose the original image into four subband images, and the image size, that is, the image resolution, becomes half of the original image. The above operation can be equal to the decomposition of the original image x using four filters ($f_{LL}$, $f_{LH}$, $f_{HL}$, $f_{HH}$),obtained four subband image $x_{LL}$, $x_{LH}$, $x_{HL}$, $x_{HH}$, namely low frequency *A*, vertical detail image *V*, horizontal detail image *H* and diagonal detail image *D*. The parameters of the filter are fixed, and they are not updated by the gradient descent operation along with the model training. The filter of Haar wavelet is shown in Eq. (1).

1 $$\begin{aligned} ^{f_{LL}=\begin{bmatrix}1&{}1 \\ 1&{}1 \end{bmatrix},\;f_{LH}=\begin{bmatrix}-1&{}-1 \\ 1&{}1 \end{bmatrix},\;f_{HL}=\begin{bmatrix}-1&{}1 \\ -1&{}1 \end{bmatrix},\;f_{HH}=\begin{bmatrix}1&{}-1 \\ -1&{}1 \end{bmatrix}} \end{aligned}$$

The input image is *x(i,j)*, *i* represents row and *j* represents column. The 2D DWT is shown below:

2 $$\begin{aligned} \left\{ \begin{array}{l} A=x_{LL}=f_{LL}\bigotimes x=x(2i-1,2j-1)+x(2i-1,2j)\\ \quad\quad +x(2i,2j-1)+x(2i,2j) \\ V=x_{LH}=f_{LH}\bigotimes x=-x(2i-1,2j-1)-x(2i-1,2j)\\ \quad\quad +x(2i,2j-1)+x(2i,2j) \\ H=x_{HL}=f_{HL}\bigotimes x=-x(2i-1,2j-1)+x(2i-1,2j)\\ \quad\quad -x(2i,2j-1)+x(2i,2j) \\ D=x_{HH}=f_{HH}\bigotimes x=x(2i-1,2j-1)-x(2i-1,2j)\\ \quad\quad -x(2i,2j-1)+x(2i,2j) \end{array} \right. \end{aligned}$$

In Eq. (2), $\bigotimes$ represents a convolution operation, the input x can be represented by a convolution operation with a different filter, and it can also be understood as downsampling with a stride of 2. Since the wavelet transform does not lose information, the wavelet transform and the inverse wavelet transform are reversible operations. For the Haar wavelet, the inverse operation can be expressed as Eq. (3).

3 $$\begin{aligned} \left\{ \begin{array}{l} x(2i-1,2j-1)=(x_{LL}-x_{LH}-x_{HL}+x_{HH})/4 \\ x(2i-1,2j)=(x_{LL}-x_{LH}+x_{HL}-x_{HH})/4 \\ x(2i,2j-1)=(x_{LL}+x_{LH}-x_{HL}-x_{HH})/4 \\ x(2i,2j)=(x_{LL}+x_{LH}+x_{HL}+x_{HH})/4 \\ \end{array} \right. \end{aligned}$$

**Figure 2:**
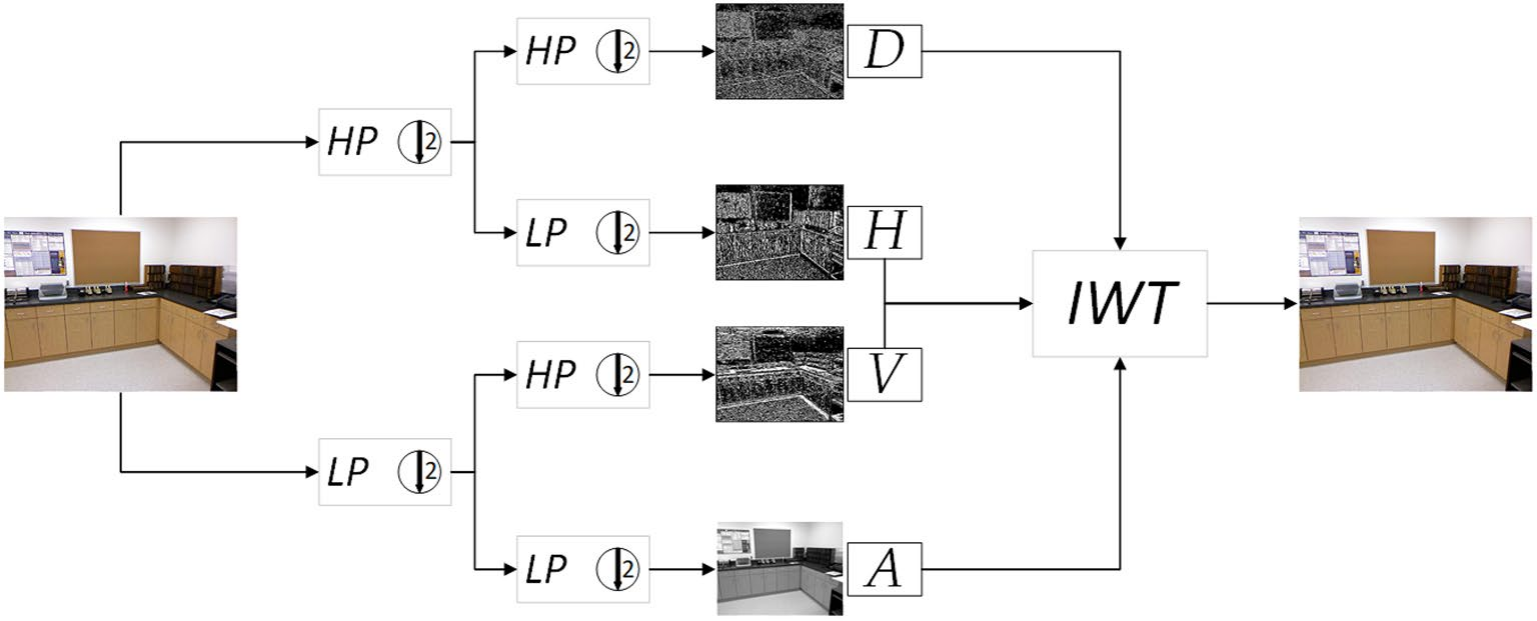
Discrete wavelet transform (DWT) and inverse wavelet inverse transform (IWT).

The original image obtains four sub-bands through DWT, or the four sub-bands obtain the original image through IWT. The process diagram is shown in Fig. 2. HP represents high-pass filtering, and LP represents lowpass filtering. $\downarrow$2 means the standard downsampling operator with factor 2.

**Figure 3:**
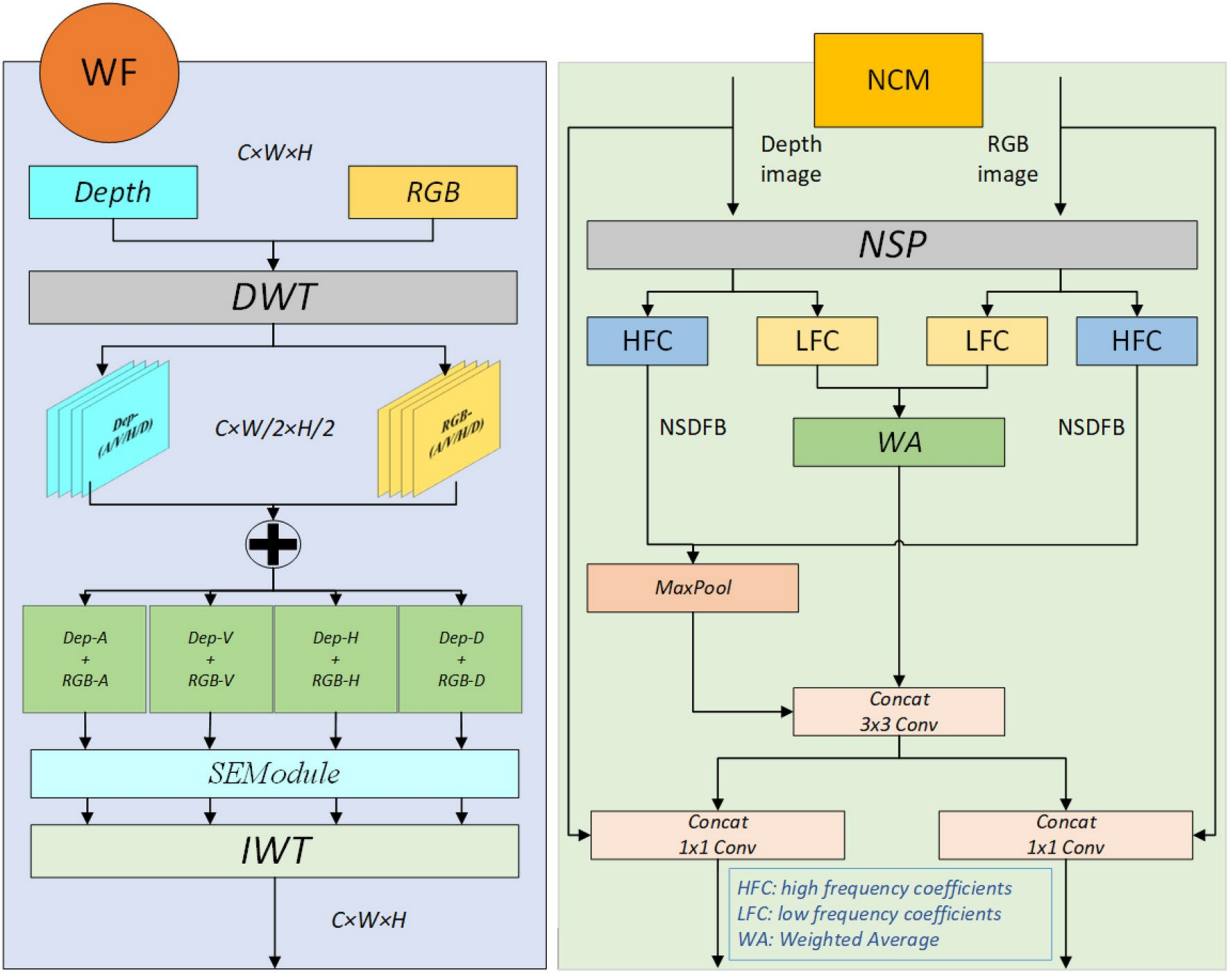
Relevant module.

The modules covered with Wavelet fusion and nonsubsampled contourlet module are shown in Fig. 3.

### Wavelet fusion module

The Wavelet transform fusion module is added to the encoder to fuse RGB color information and depth information. The two feature information are first decomposed into four different subbands (A, V, H, D) by DWT, and then the corresponding subbands of RGB color image and depth image are added. Through the attention module (SEModule)^39^, the output is finally fused by the inverse wavelet transform.

### DWT block

When the image information enters the block, it is decomposed into four subbands through DWT, and then it concats the four subbands. The resolution of the obtained feature information becomes half of the input, and the number of channels become 4 times, as a result the number of channels becomes the same as the input channel value through a 1 × 1 convolution operation.

### Nonsubsampled contourlet module

**Figure 4:**
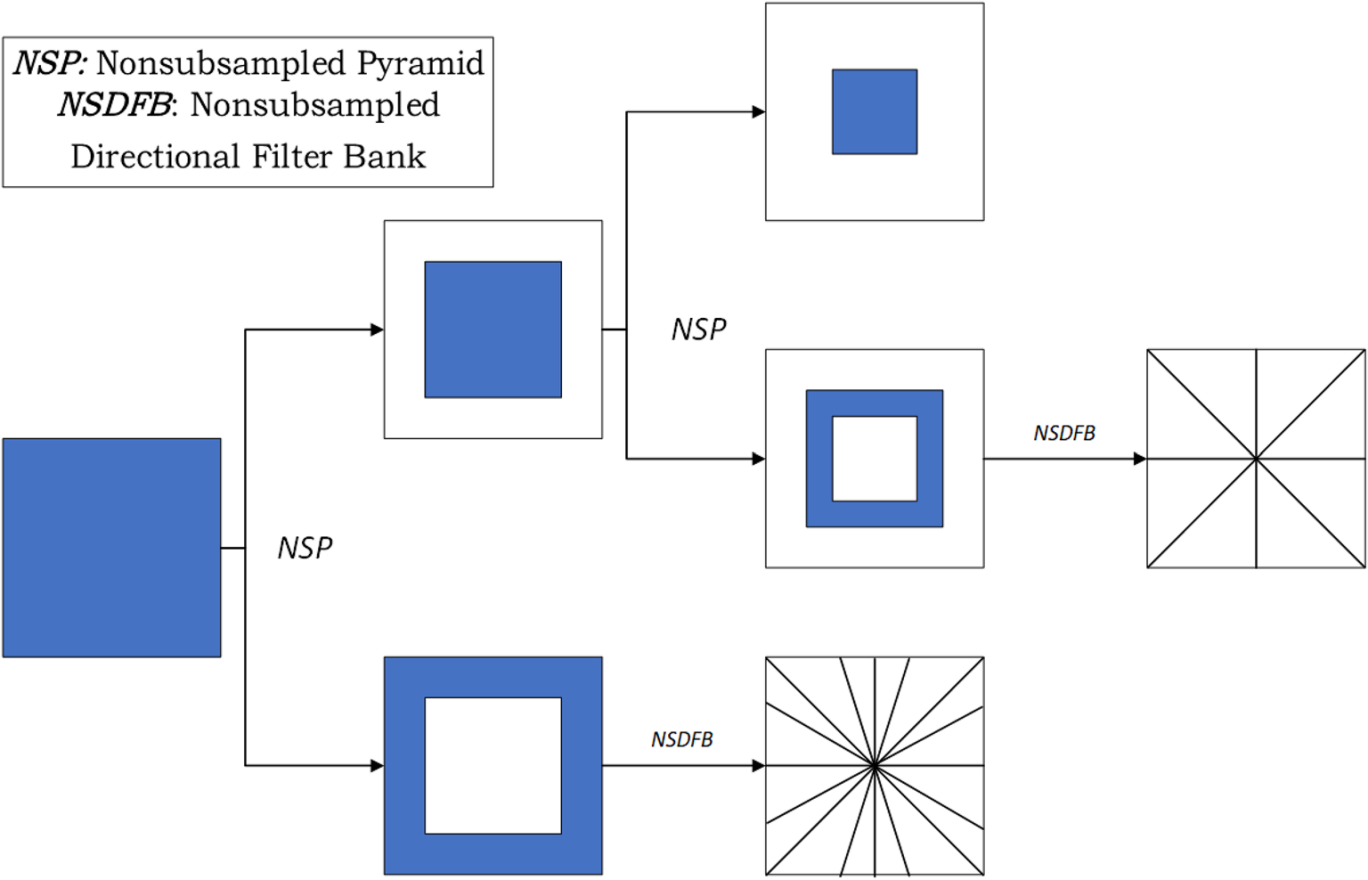
Structure of nonsubsampled contourlet transform.

The nonsubsampled contourlet module (NCM) is designed to replace the pooling operation in ResNet. We find that the pooling operation in the neural network will lose the corresponding information in the downsampling process more or less, and the features learned by a single convolution operation are limited. At the same time, another disadvantage of pooling operation is that the neural network will lose translation invariance. To solve the above problems, we designed NCM, combined with convolution operation, The Nonsubsampled Pyramid (NSP) and Nonsubsampled Directional Filter Bank (NSDFB) preserve the multi-scale properties and directionality of the original image. This is particularly important for the fusion of depth information. Nonsubsampled Contourlet transform as shown in Fig. 4.

The decomposition scale of the image is defined as *D*. NSP can obtain the subband information with the number of $D+1$ by decomposition. The decomposition formula is as follows:

4 $$\begin{aligned} \begin{matrix} R=\sum _{i=0}^{i-1} 2^{k_{l}} \end{matrix} \end{aligned}$$

The number of decomposition levels is denoted by *i*, and the number of decompositions is an integer power of two.The Nonsubsampled contourlet module is shown in the following equation:

5 $$\begin{aligned} \begin{matrix} I=i_{j}+\sum _{i=0}^{i-1} \sum _{k=0}^{l_{i}} d_{j, k} \end{matrix} \end{aligned}$$

The low-frequency self-band information decomposed in the *j* scale direction is denoted by $i_j$, and the subband information in the *k* direction is denoted by $d_{j, k}$

### Multiple pyramid module

**Figure 5:**
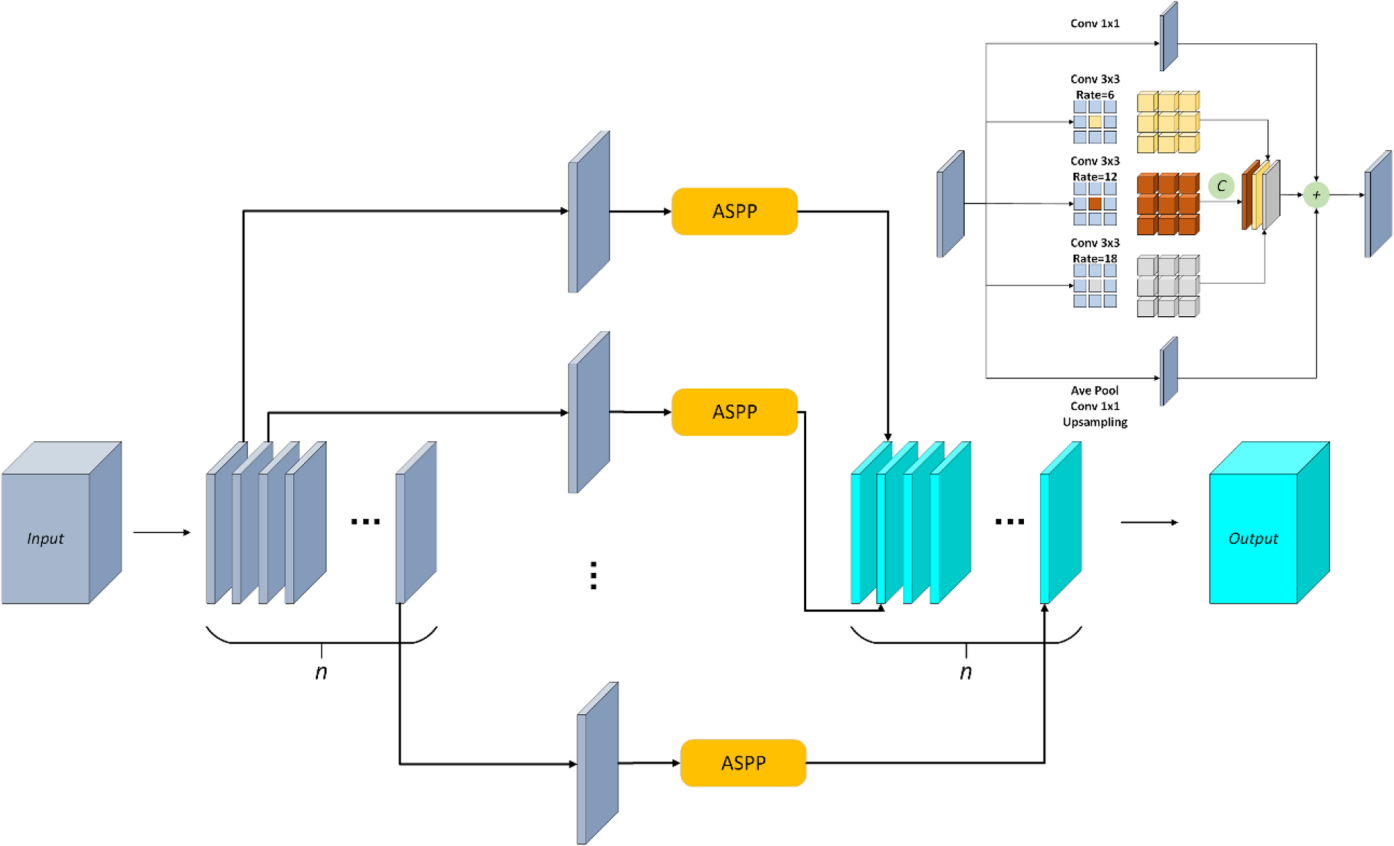
Structure of multiple pyramid module.

Atrous Spatial Pyramid Pooling (ASPP) can expand the receptive field, strengthen the contact different context information, but as a result of dilated convolution is a layer of the neighboring pixels from independent subset is obtained by convolution operation, lack of dependence on each other, and the convolution results from a layer of the independent set of local information is missing, so we designed the Multiple pyramid module, as shown in Fig. 5. Firstly, channel splitting is carried out for the current input feature map $F_{i n} \in {\mathbb {R}}^{H x W x C}$, which is divided into N parts. Then, each part $F_{i n} \in {\mathbb {R}}^{H x W x \frac{C}{n}}$ is subjected to Atrous Spatial Pyramid Pooling (ASPP), and the obtained feature map is splicing. Thus, the situation of ignoring the internal information relation while processing a single feature map is avoided, and the global context information feature is fully extracted. ASPP includes a 1x1 convolution, three 3x3 convolution which dilated rate respectively for 6, 12, 18 and the average pooling, we use concatenation operation on empty part of the convolution operation, keep more original location information, using the way of combined operation 1x1 convolution and average characteristics after the pooling, which ensure every dimension contains more information. The dilated convolution operation is as follows:

6 $$\begin{aligned} \begin{matrix} y(i, j)=\sum _{u=0}^{H} \sum _{v=0}^{W} x(i+a r \times u, j+a r \times v) \times Weight(u, v) \end{matrix} \end{aligned}$$

where *H*,*W* represents the length and width of the input image, *x(i,j)* represents the pixel value at the position *(i,j)*, AR represents the cavity rate, y represents the output, *(u,v)* represents the central coordinate of the convolution kernel, and *Weight* represents the global average pooling of the weight of the convolution kernel at the corresponding position, as shown below:

7 $$\begin{aligned} \begin{matrix} X_{(i, j)}^{\prime }=\frac{1}{H \times W} \sum _{i=1}^{H} \sum _{j=1}^{W} X_{(i, j)} \end{matrix} \end{aligned}$$

$X_{(i, j)}$ represents the pixel value of the input image at position *(i,j)*,$X_{(i, j)}^{\prime }$ represents the pixel value of the output feature map.

### Encoder

Resnet-50 is adopted as the backbone network of the encoder, and there are four ResNet blocks in each of the two branches. At the same time, for high-frequency information such as prominent edge contour represented in the depth map, as the depth of the encoder in the network gradually deepens, some edge contour features may be lost. Therefore, we add a discrete wavelet transform module to replace the pooling operation in the original ResNet to retain more information of different frequencies, and fuse the high-frequency edge information obtained from the depth image into the RGB color image through the wavelet transform fusion module.

### Decoder

**Figure 6:**
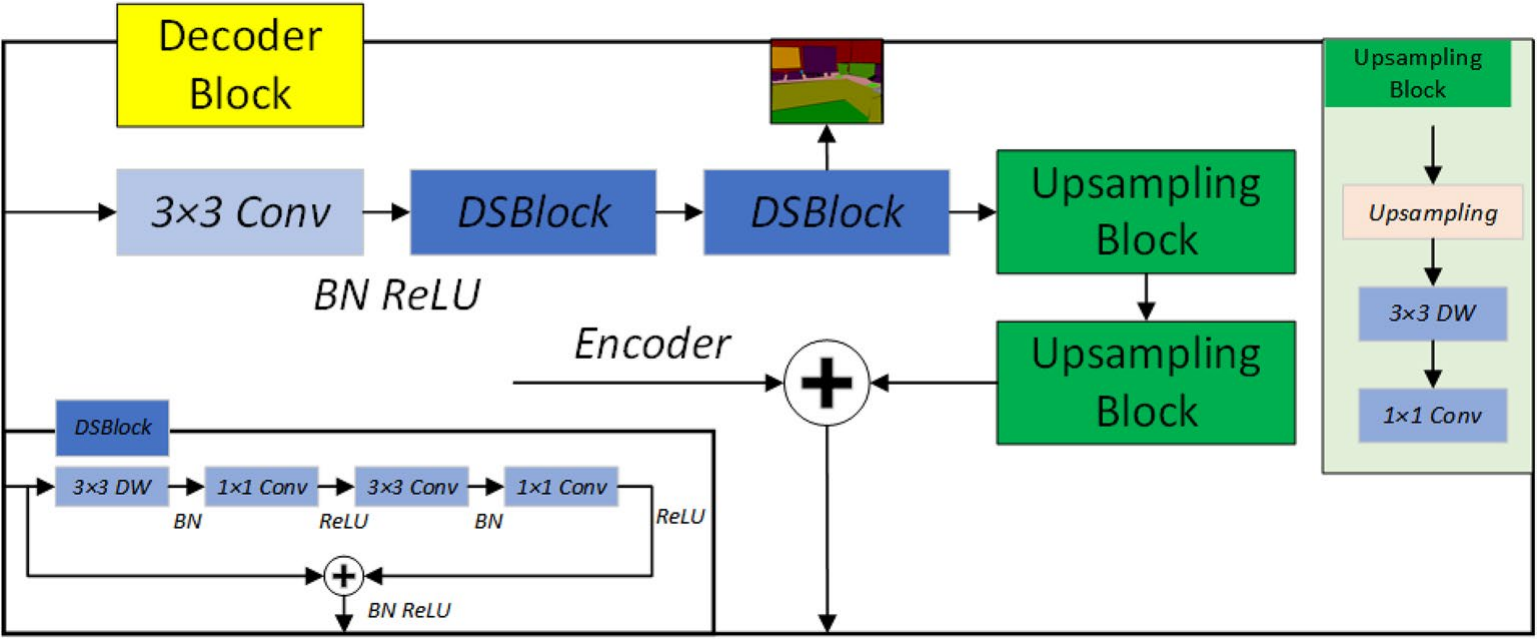
Decoder module.

The decoder in this paper is composed of three decoder modules. The decoder module is shown in Fig. 6., and the number of channels is reduced from 2048 with the resolution increasing through convolution operation. In addition, we integrated deep separable convolution^40^. Deep separable convolution can reduce the number of parameters and save the computational cost. Through these operations, the accuracy and efficiency of semantic segmentation can be further improved. Finally, the resolution is increased by nearest neighbor upsampling, and the feature information is integrated by depth-separable convolution. It is difficult to avoid information loss during the up-sampling process of the decoder. We use 1 × 1 convolution operation to integrate the fused features of the encoder RGB-D through decoder hopping connection, and restore the image resolution through two up-sampling modules after three decoder modules. At the same time, we designed a multi-level Loss function, adding an output after each decoder module, inputting outputs of different resolutions and final results to the end of the network and finally obtaining the Loss function. The Loss function selects the cross-entropy function, as shown in Eq. (6):

8 $$\begin{aligned} \begin{matrix} {\text {Loss}}(x, {\text {class}})=\frac{1}{N} \sum _{i}-\log \left( \frac{\exp (x[{\text {class}}])}{\sum _{j} \exp (x[j])}\right) \end{matrix} \end{aligned}$$

where class represents the category of pixel *i* in the label graph, *X* represents the score of pixel *i*, and *N* represents the total resolution of the output image. Since there are also three decoder module outputs, the total loss function is the sum of four partial loss functions, and because different outputs have different resolutions, we assign different weights according to the size of the resolution, and the ratio is 1:2:3:4.

Algorithm 1 shows the details of the proposed method.
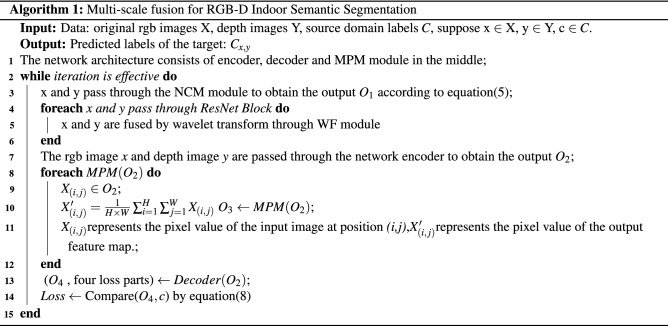


## Experiments and results

In this section, we first introduce the parameters and environment settings of the experiment, and then introduce the dataset and the selected evaluation metrics. Then, ablation experiments are designed for each module, such as the fusion module and the multiple pyramid module, to verify the effect of the designed module and the rationality of the hyperparameters. Finally, the corresponding experimental results are displayed on two public datasets.

### Environment of experiment

In order to verify the performance of the proposed method, this paper conducts experiments on two public datasets, NYUV2 and SUN-RGBD datasets. RGB color images and depth images are input at the same time. The environment of this experiment is with Intel I7-12700 CPU and 64G memory and an NVIDIA GeForce RTX 3090 graphics card. The model is trained by gradient descent method, and the deep learning framework of PyTorch is selected at the same time. The learning rate is set to $5e-4$, and the optimizer selects Range optimizer to update the parameters. At the same time, transfer learning^41^ is used to take Resnet-50 pre-training weight on Imagenet dataset as the initial weight, which effectively accelerates the training speed of the entire network. The epoch of training is set to 300, and save the weight with best result of validation set.

### Datasets and performance measures

We design experiments on two public datasets, NYUV2 and SUN RGB-D, the NYUv2 dataset contains 1449 densely annotated RGB image and depth image pairs, including a total of 464 different scenes. In addition, the dataset contains 35064 different objects, covering 894 different object categories in 1449 images. Our work adopts the standard division strategy of NYUv2 dataset: 795 images are used as the train set, and the remaining 654 images are used as the test set. We also used the common 40-class label setting SUNRGB-D dataset has 37 categories, including 10335 RGBD images with densely labeled semantic labels. The dataset consists of several existing small-scale datasets and some RGB-D images taken by the authors themselves, including 3784 images (taken with Kinect v2), 1159 images (taken with Intel RealSense), 1449 images of NYU v2 dataset (taken with Kinect v1), 554 images carefully selected from Berkeley B3DO dataset (taken with Kinect v1), and 3389 images selected from SUN3D dataset (taken with Asus Xtion). It uses 5050 images as the testset and 5285 images as the trainset. The ablation experiment and semantic segmentation effect test in our work are based on NYUv2, and the entire network input resolution is 480 × 640.

Three semantic segmentation evaluation indexes were used to evaluate the experimental results in this paper, including Pixel Accuracy (PA), Mean Pixel Accuracy, MPA and Mean Intersection over Union, and MIoU. PA is the ratio of the number of correctly classified pixels and all pixels in a picture, equation is defined as follows:

9 $$\begin{aligned} \begin{matrix} P A=\frac{\sum _{i=0}^{k} p_{i i}}{\sum _{i=0}^{k} \sum _{i=0}^{k} p_{i j}} \end{matrix} \end{aligned}$$

where $p_{i i}$ represents the number of pixels that are correctly classified, and $p_{i j}$ represents the number of pixels that belong to class *i* but are predicted to be class *j*.

MPA is the average value of the ratio between the number of correctly classified pixels in each class and all pixel points, which is defined as follows:

10 $$\begin{aligned} \begin{matrix} M P A=\frac{1}{k} \times \sum _{i=0}^{k} \frac{p_{i i}}{\sum _{j=0}^{k} p_{i j}} \end{matrix} \end{aligned}$$

*k* indicates the number of categories.

MIoU is the average value of the ratio of the intersection and union of two sets of real value and predicted value, which is defined as follows Eq. (9):

11 $$\begin{aligned} \begin{matrix} M I o U=\frac{1}{k} \times \sum _{i=0}^{k} \frac{p_{i i}}{\sum _{j=0}^{k} p_{i j}+\sum _{j=0}^{k} p_{j i}-p_{i i}} \end{matrix} \end{aligned}$$

### Ablation experiments

In this subsection, we design a series of experiments to evaluate the effectiveness of each module, and the ablation experiments are performed on the NYUv2 dataset.

#### Ablation experiment of fusion module

In this section, we design multiple experiments to compare the wavelet transform fusion module with other fusion methods. RedNet adds depth image and RGB image directly. ACNet extracts image features by balancing the distribution of features through ACM (Attention Complementary Modules) and adding a third branch. The SE (Squeeze and Excitation) module is used first for feature extraction, followed by the additive fusion. Except for the fusion mode, the other modules remain unchanged, and the experimental results are shown in the Table 1. According to the experimental results, it can be seen that the Wavelet transform fusion has a good performance in segmentation accuracy and inference speed, thanks to the characteristics of the wavelet transform without additional computation and the effective retention of the contour details of the original image. In the fusion part, different from other methods, the original image is decomposed into four sub-bands through wavelet transform and inverse wavelet transform, so that each sub-band can retain its own frequency characteristics, and the feature performance is strengthened through the attention mechanism, and then the performance in the depth map and color map is highlighted.

**Table 1:** Ablation experiment of fusion module.

Method	Attention	MIoU (%)	FPS
Baseline + RedNet	None	48.30	31
Baseline + ACNet	ACM	49.28	17
Baseline + ESANet	SE	51.05	21
MSFNet	SE	52.23	24.7

#### Ablation experiment of nonsubsampled contourlet transform module

**Figure 7:**
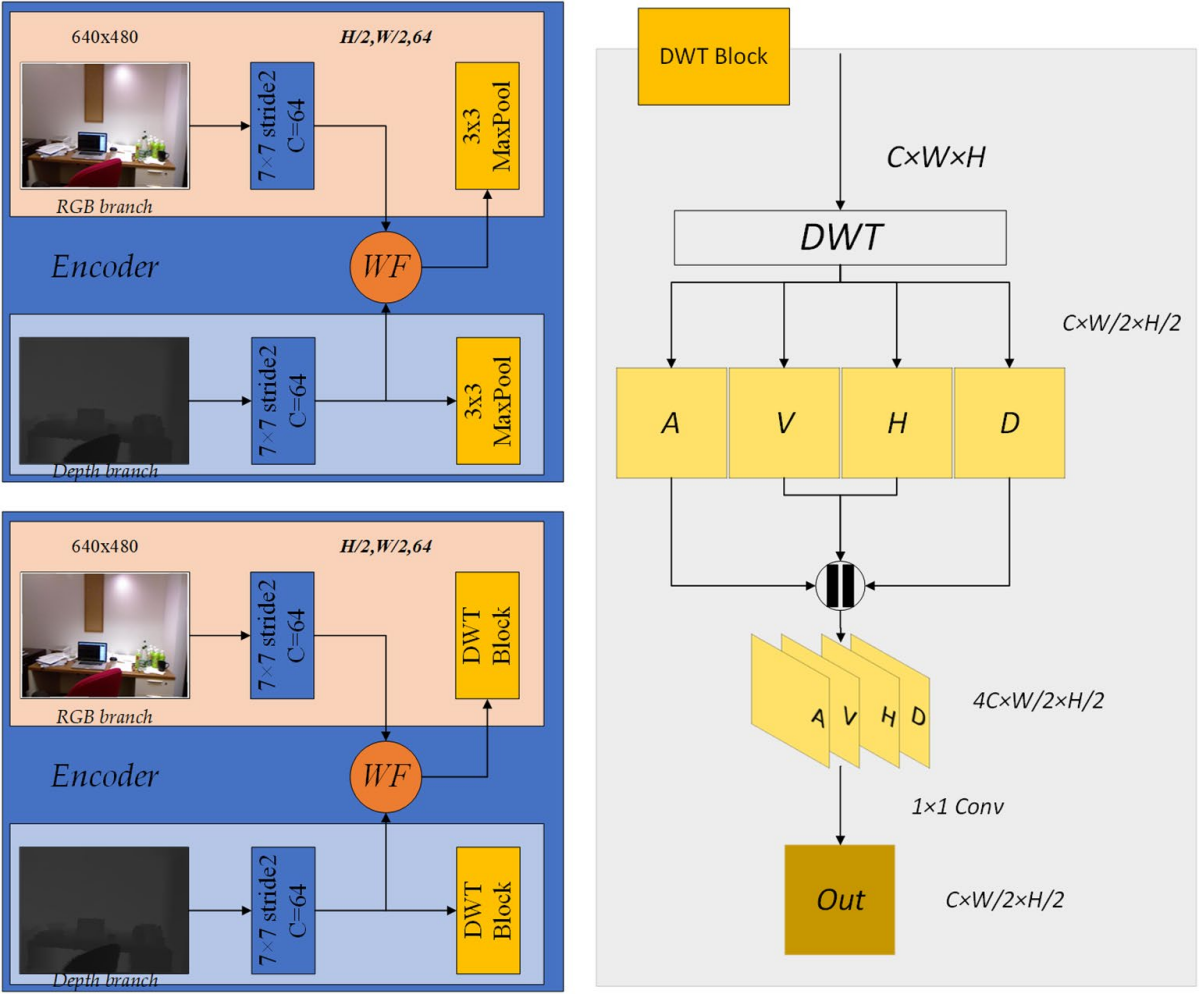
Compare with pooling operations.

In order to verify the effectiveness of the Nonsubsampled contourlet transform module we designed, we designed A set of comparison experiments. Model A used ResNet original pooling operation, namely 3x3 maximum pooling, and used wavelet transform to fuse the feature maps of the two branches. Model B uses wavelet transform to concatenate the four subbands to obtain the down-sampling result. The operation process is shown in Fig. 7, and the result is shown in Table 2.

**Table 2:** Ablation experiment of nonsubsampled contourlet transform module.

Method	Pooling operation	MIoU (%)
Model A	3 × 3 Max pool	51.66
Model B	DWT block	51.85
MSFNet	NCM	52.23

It can be seen from the results that the segmentation accuracy has been slightly improved after adding NCM. Although the wavelet transform module has also been improved, there are still some deficiencies in the extraction and retention of edge contour features in the shallow layer of the network due to the lack of translation sensitivity and multi-direction recognition by simply using wavelet transform. NCM retains the multi-scale characteristics and directionality of the original image more effectively, Preserving the above features in the thousand-layer part of the neural network is particularly important for the whole training process.

#### Ablation experiment of multiple pyramid module

**Figure 8:**
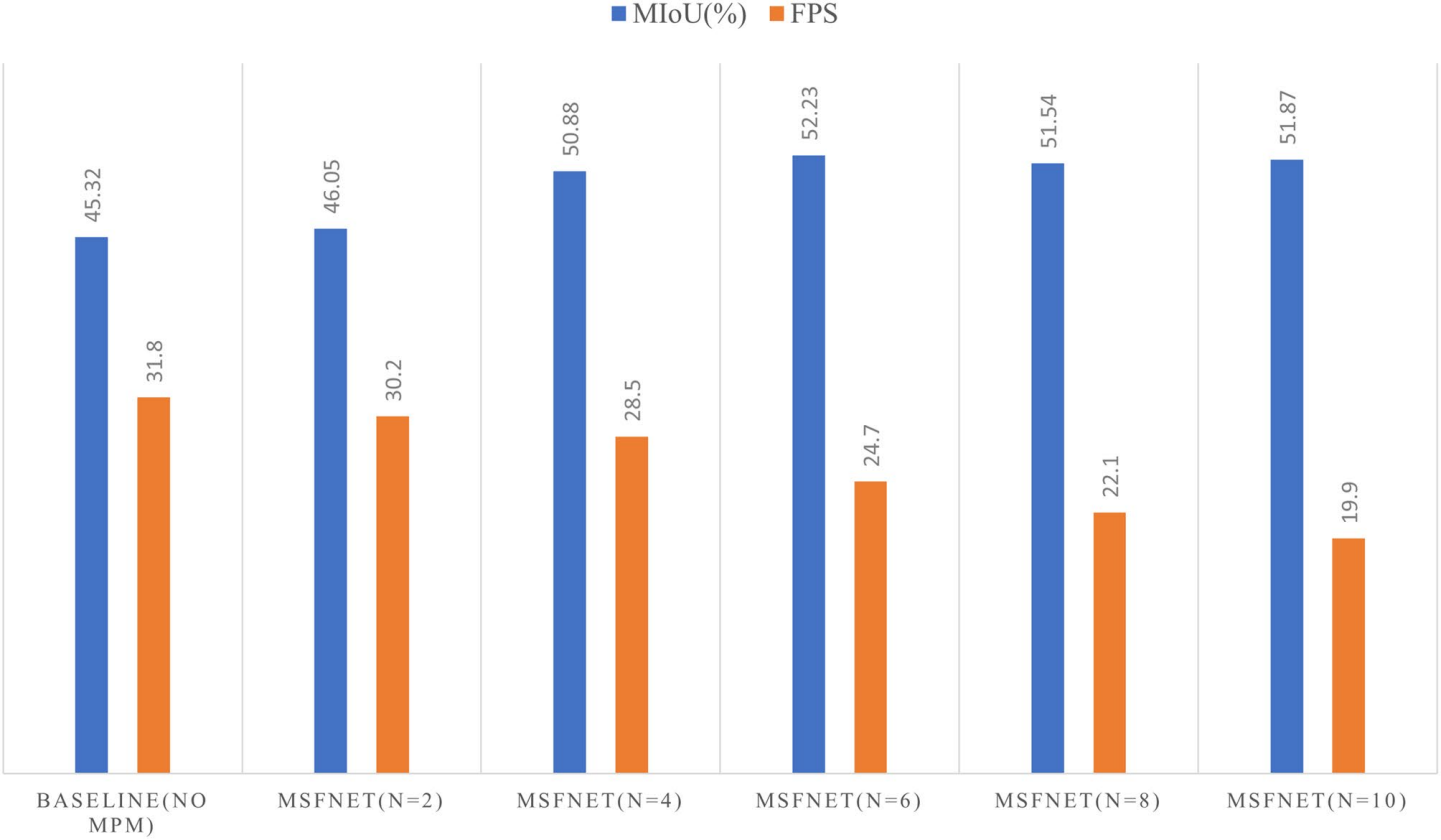
Result of segmentation.

The hyperparameter experiments on the multiple pyramid module are tested on the NYUv2 dataset. In this section, we study the influence of hyperparameter n on the performance of the fusion module. N means that the feature map is divided into several parts according to the number of channels. Five groups of experiments with N of 2, 4, 6, 8 and 10 are designed, and the evaluation indexes are mIoU and inference speed, the result is shown in Fig. 8.

As can be seen from the above result, with the gradual increase of N, mIoU also increases, reaching 52.23$\%$ when N is 6. However, the intersection ratio fluctuates with the subsequent increase of N, and the inference speed gradually decreases. On this basis, we select N = 6 as the hyperparameter of the multi-pyramid module according to the inference speed and segmentation accuracy.

In order to verify the effectiveness of the multi-pyramid module we designed, we compared some Fusion methods horizontally, such as DenseASPP^42^, ASPP^43^ and SAPF (Scale-aware Pyramid Fusion)^44^. We replaced the Fusion module with different Fusion methods. Other modules remain unchanged, and the experimental results are shown in Table 3 benefit by the multiple pyramid module designed by us, the receptive field is not only increased in a single dimension, but also the feature information connection between contexts is strengthened through the splitting and stitching of channels, so as to improve the segmentation accuracy.

**Table 3:** Ablation experiment of context module.

Method	Params/M	MIoU (%)	FPS
Baseline + DenseASPP	76.25	48.86	15.3
Baseline + ASPP	44.35	48.80	22.5
Baseline + SAPF	71.18	47.22	18.3
MSFNet	48.64	52.23	24.7

#### Ablation experiments of each module

This section analyzes the influence of the added wavelet transform modules (wavelet fusion module, wavelet transform replacement pooling module and wavelet transform connection module) on the semantic segmentation results. In order to analyze the influence of different modules, we constructed the four kinds of models, are respectively the ABCD, in A model, we don’t add any module structure of encoder and decoder, fusion form is directly convolution operation, we only add in the model B wavelet fusion module, in C model, we added on the basis of the model B replace DWT pooling operation module, In model D, we add a context connection module on the basis of model B. Table 4 summarizes the experimental results of each model. Can be seen from the results of B add wavelet transform fusion module can effectively improve the performance of the semantic segmentation can be seen from the CD in the WTC module for network more obvious indicators of ascension, this is because the wavelet transform for the outline of encoder and decoder joint can retain more details information, such as improving the precision of semantic segmentation. Compared with Model B, adding NCM module and MPM module improves the segmentation accuracy of model B by 0.5% and 2.2% respectively. The NCM module retains the frequency and orientation features of the image, and the MPM module plays the role of preserving the context information at the encoder-decoder junction transition stage, and fuses multi-scale features.

**Table 4:** Ablation Experiments.

Model	WT-fusion	NCM	MPM	PA (%)	MPA (%)	MIoU (%)
Model A				73.9	58.8	45.3
Model B	$\surd$			74.6	61.0	49.48
Model C	$\surd$	$\surd$		75.9	61.3	49.98
Model D	$\surd$		$\surd$	76.3	62.8	51.66
WTNet	$\surd$	$\surd$	$\surd$	77.8	64.2	52.23

### Results on NYUv2 and SUN-RGBD datasets

We tested our network model on NYUv2 and SUNRGBD datasets and compared it with existing network algorithms. The experimental results are shown in Table 5. The experimental results are shown in Table 6. Experimental results show that the proposed algorithm improves the pixel accuracy, the average pixel accuracy and the average intersection point ratio. The specific values on NYUv2 dataset are 77.8, 64.2 and 52.23$\%$, and those on SUN-RGBD dataset are 83.6, 62.4 and 50.32$\%$. Compared with the current SOTA algorithm, the three evaluation indexes have been improved. We believe that this is due to the combination of the wavelet transform RGB-D fusion, which retains more details and makes the two have better fusion effect. While at the same time only using the ResNet-50 coding structure, instead of using a network of deeper encoding but by replacing pooling layer as well as the design of the encoder and decoder connection module, wavelet transform to the edge of the target object information more accurate, at the same time, make the whole network is not deep structure of the encoder can get very good segmentation effect, the experimental results are shown in Table 5.

**Table 5:** Results on Datasets.

Model	PA (%)		MPA (%)		MIoU (%)	
	NYUv2	SUN- RGBD	NYUv2	SUN- RGBD	NYUv2	SUN- RGBD
RDF (2017)^45^	76.0	81.5	62.8	60.1	50.1	47.7
ACNet (2019)^27^	–	–	–	–	48.3	48.1
CTNet (2019)^46^	76.3	82.4	_	–	50.6	48.5
TSNet (2020)^47^	73.5	_	59.6	–	46.1	_
SGNet (2021)^30^	76.1	82.0	62.7	–	50.7	48.6
ESANet (2021)^37^	–	–	–	–	51.58	48.31
EMSANet (2022)^48^	–	–	–	–	53.34	48.47
FRNet (2022)^49^	77.6	87.4	66.5	62.2	53.6	51.8
MSFNet (ours)	**77.8**	**83.6**	**64.2**	**62.4**	**52.23**	**50.32**

Experimental results show that our MSFNet has good performance on different datasets, which indicates that it can adapt to multiple categories and scenarios. Meanwhile, we test the parameter size and inference speed of MSFNet on NYUv2 dataset. The experiment was deployed on an NVIDIA 1080Ti. The results are shown in Table 6. It can be seen from the results that the wavelet transform module we designed only needs a small amount of extra computation, and still can achieve the speed of real-time inference.

**Table 6:** Inference Speed test on NYUv2 Dataset.

Model	Backbone	MIoU (%)	Param (M)	FPS
RDF (2017)^45^	2ResNet101	49.1	169.1	11
ACNet (2019)^27^	2ResNet50	48.3	116.6	18
SGNet (2021)^30^	2ResNet50	47.7	39.3	39
ESANet (2021)^37^	2ResNet50	50.53	54.46	22.6
EMSANet (2022)^48^	2ResNet34	53.34	–	24.5
MSFNet (ours)	2ResNet50	52.23	48.64	24.7

**Figure 9:**
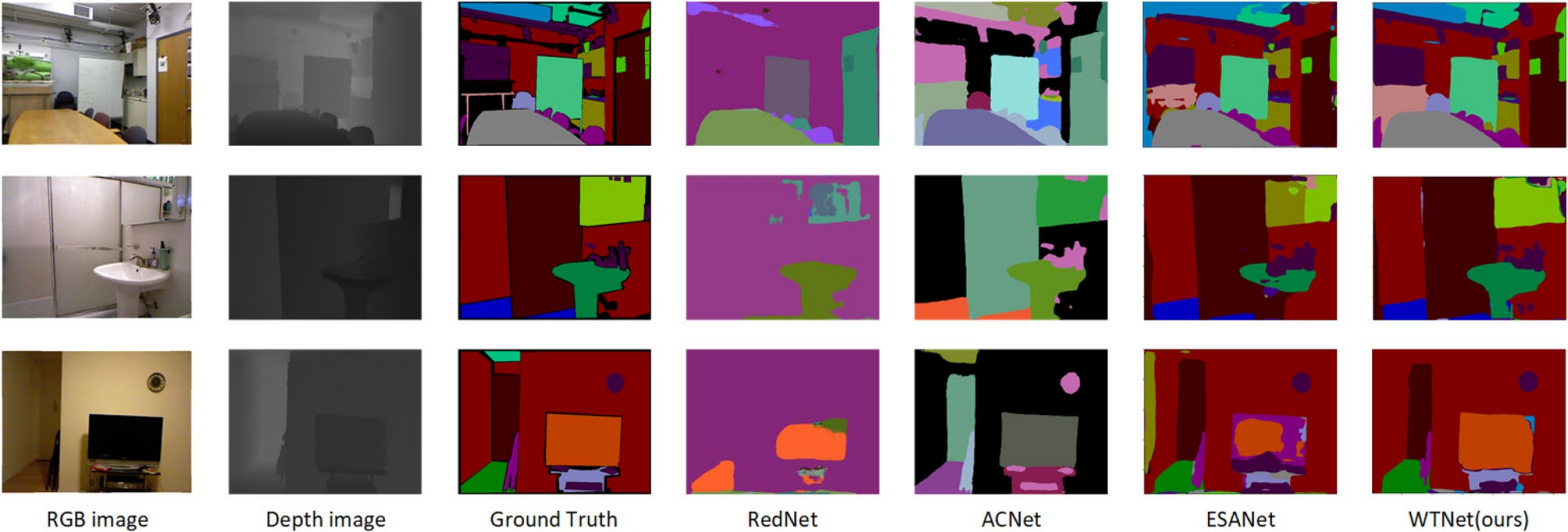
Result of segmentation.

**Figure 10:**
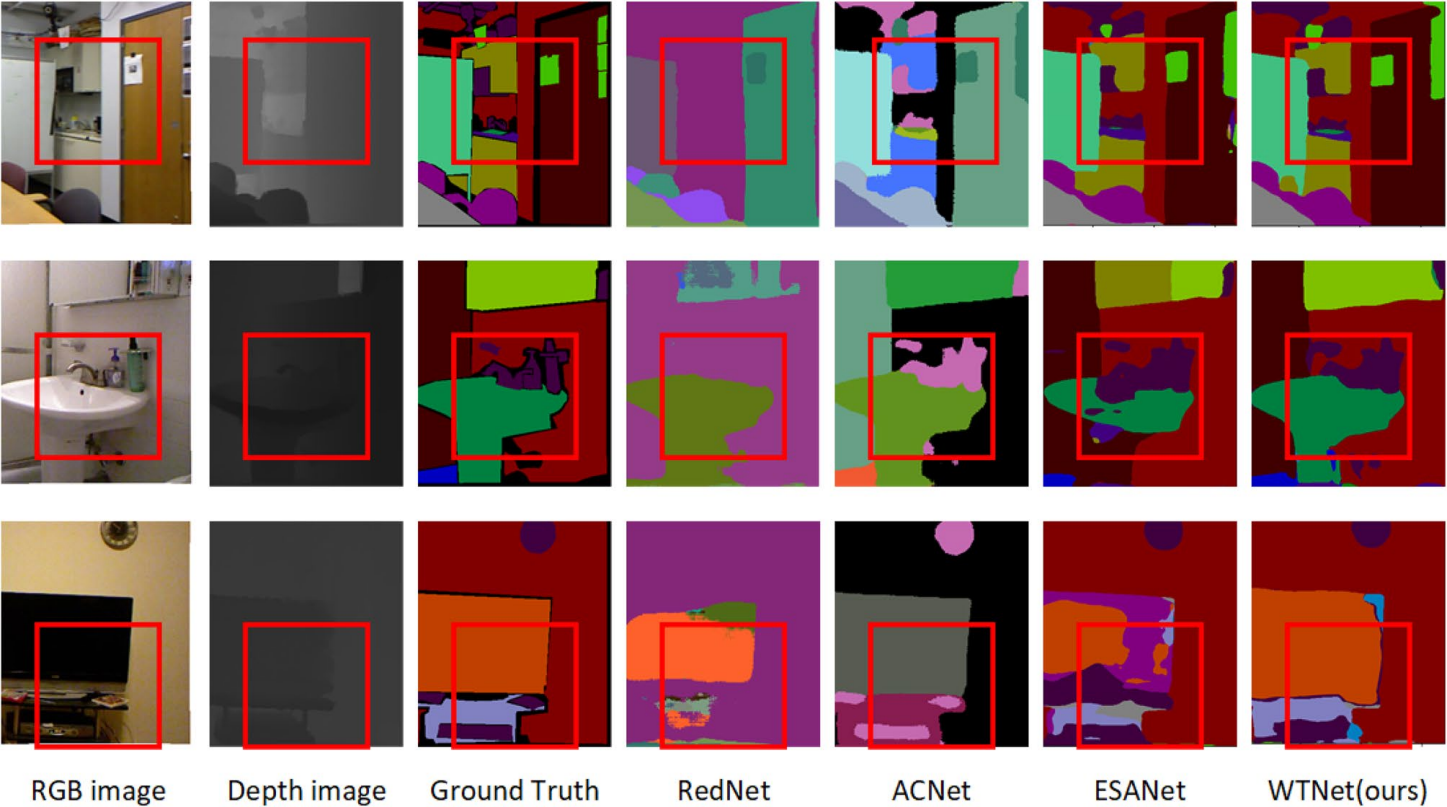
Detail of Segmentation result.

At the same time, we compare the semantic segmentation results with RedNet, ACNet and ESANet^37^ networks. The residual module in RedNet is used as a basic building block in encoder and decoder to construct a fusion structure and propose a pyramid supervised training scheme. ACNet uses independent branches based on ResNet to fuse RGB features and depth features, and finally obtains segmentation results after multiple up-sampling. ESANet also adopts ResNet as the backbone network, adopts direct additive fusion in the fusion part, and adds a module similar to pyramid pooling at the encoder-decoder connection^50^. The segmentation results of each network are shown in Fig. 9, from which it can be seen that more details are preserved through the wavelet transform module. The segmentation results not only guarantee the contour details of the larger object, but also retain the information of some small object. The segmentation details are shown in Fig. 10.

From the segmentation details, it can be seen that thanks to the wavelet fusion and other modules designed by us, the edge contour information is relatively complete. For example, the wall and door frame details in the first line are smoother, and the edge contour details of the wash basin in the second line are also more abundant. In the third row, although the TV edge is not as good as ACNet segmentation, the details in the TV cabinet are more complete.

## Conclusion

In this study, we propose an RGB-D indoor semantic segmentation network based on multi scale fusion. Through the ResNet-50 as backbone of color image and the depth image encoder, add wavelet transform fusion module, and replace the pooling DWT wavelet module operation, and design a multiple pyramid module to aggregate multi-scale information and context global information, combined with low frequency and high frequency information fully, retains the outline details of the object, obtain more accurate information, and use nonsubsampled contour wave transform replaces the pooling operation. To further optimize the network segmentation effect, the encoder-decoder is connected by hop connection, and three side output supervised loss functions are added simultaneously. Experimental results on two datasets show that the performance of the proposed MSFNet is better than the current models, and the speed also meets the requirements of real-time inference, which provides a new method and idea for RGB-D indoor scene segmentation.

## Data Availability

The datasets generated and analysed during the current study are available in the NYUv2 repository and SUNRGB-D repository, [https://cs.nyu.edu/silberman/datasets/nyu_depth_v2.html] and [https://rgbd.cs.princeton.edu/].
